# CRISPR-*cas3* of *Salmonella* Upregulates Bacterial Biofilm Formation and Virulence to Host Cells by Targeting Quorum-Sensing Systems

**DOI:** 10.3390/pathogens9010053

**Published:** 2020-01-10

**Authors:** Luqing Cui, Xiangru Wang, Deyu Huang, Yue Zhao, Jiawei Feng, Qirong Lu, Qinqin Pu, Yulian Wang, Guyue Cheng, Min Wu, Menghong Dai

**Affiliations:** 1 The Cooperative Innovation Center for Sustainable Pig Production, Huazhong Agricultural University, Wuhan 430070, China; cuiluqing@webmail.hzau.edu.cn (L.C.); wangxr228@mail.hzau.edu.cn (X.W.); yue1226@webmail.hzau.edu.cn (Y.Z.); fjw@webmail.hzau.edu.cn (J.F.); 2 Department of Biomedical Sciences, University of North Dakota School of Medicine and Health Sciences, Grand Forks, ND 58203, USA; qinqin.pu@und.edu; 3 MOA Key Laboratory of Food Safety Evaluation/National Reference Laboratory of Veterinary Drug Residue (HZAU), Huazhong Agricultural University, Wuhan 430070, China; huangdeyu@webmail.hzau.edu.cn (D.H.); lqrong@webmail.hzau.edu.cn (Q.L.); wangyulian@mail.hzau.edu.cn (Y.W.); chengguyue@mail.hzau.edu.cn (G.C.)

**Keywords:** type I-E CRISPR-Cas system, *cas3*, *Salmonella* virulence, RNA-Seq, quorum sensing, biofilm formation, SPI-1-T3SS

## Abstract

*Salmonella* is recognized as one of the most common microbial pathogens worldwide. The bacterium contains the clustered regularly interspaced short palindromic repeats (CRISPR)-CRISPR-associated (Cas) systems, providing adaptive immunity against invading foreign nucleic acids. Previous studies suggested that certain bacteria employ the Cas proteins of CRISPR-Cas systems to target their own genes, which also alters the virulence during invasion of mammals. However, whether CRISPR-Cas systems in *Salmonella* have similar functions during bacterial invasion of host cells remains unknown. Here, we systematically analyzed the genes that are regulated by Cas3 in a type I-E CRISPR-Cas system and the virulence changes due to the deletion of *cas3* in *Salmonella enterica* serovar Enteritidis. Compared to the *cas3* gene wild-type (*cas3* WT) *Salmonella* strain, *cas3* deletion upregulated the *lsrFGBE* genes in *lsr* (*luxS regulated*) operon related to quorum sensing (QS) and downregulated biofilm-forming-related genes and *Salmonella* pathogenicity island 1 (SPI-1) genes related to the type three secretion system (T3SS). Consistently, the biofilm formation ability was downregulated in the *cas3* deletion mutant (Δ*cas3*). The bacterial invasive and intracellular capacity of Δ*cas3* to host cells was also reduced, thereby increasing the survival of infected host cells and live chickens. By the transcriptome-wide screen (RNA-Seq), we found that the *cas3* gene impacts a series of genes related to QS, the flagellum, and SPI-1-T3SS system, thereby altering the virulence phenotypes. As QS SPI-1-T3SS and CRISPR-Cas systems are widely distributed in the bacteria kingdom, our findings extend our understanding of virulence regulation and pathogenicity in mammalian hosts for *Salmonella* and potentially other bacteria.

## 1. Introduction

**Figure 1:**
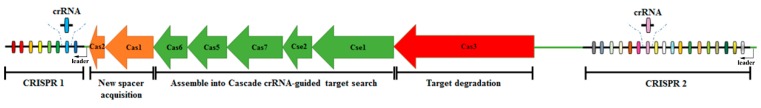
Schematic of the *Salmonella* type I-E clustered interspaced short palindromic repeats (CRISPR)-CRISPR-associated (Cas) operon. *Salmonella* has two CRISPR loci, CRISPR1 and CRISPR2, both encoded on the minus strand. There are eight *cas* genes that are located upstream of CRISPR1, shown as colored boxed arrows. Type I system signature gene, *cas3*, is shown (red). The *cas1* and *cas2* genes are universal, and present in all CRISPR-Cas systems (orange). The remaining *cas* genes are type I-E-dependent (green). The CRISPR locus comprises a leader region and repeats (black cross lines) that are separated by unique spacers (vertical colored lines). crRNA (CRISPR RNA) consists of a spacer flanked by partial repeats.

*Salmonella* is a significant pathogen for humans and animals. It not only causes a variety of diseases in livestock and poultry but also causes food poisoning in humans [1]. Among the foods that cause *Salmonella* poisoning, more than 90% are meat and other animal products [2,3]. It is a highly diverse species comprising of two species divided into six subspecies and over 2500 serovars [4]. Statistics show that *Salmonella enterica* serovar Enteritidis (*S.* Enteritidis), *S.* Choleraesuis, and *S.* Typhimurium are the major pathogenic bacteria that contaminate animal products and cause human *Salmonella* poisoning. These bacteria cause significant harm to human health and livestock development [5]. In this study, the *Salmonella* strain SE211 serotype is identified as *S.* Enteritidis, one of the dominant serotypes. SE211 is characterized as a highly pathogenic *Salmonella* harboring a type I-E clustered interspaced short palindromic repeats (CRISPR)-CRISPR-associated (Cas) system. The system comprises two major molecular constituents: A set of *cas* genes and CRISPR arrays (Figure 1). CRISPR arrays have been widely used in the serotyping of *Salmonella* according to the variety in the spacers [6,7], and it was shown that the results from CRISPR typing had a good correspondence with whole genome sequence typing [8,9]. Furthermore, CRISPR-multi-virulence-locus sequence typing (MVLST) has been frequently used to subtype *Salmonella* serovars [10,11]. The conserved genetic organization of the *cas* genes in some *Salmonella* serovars is consistent with its biological function in these bacteria [12,13,14]. In addition, some reports found that in *S.* Typhi, this system consists of five transcriptional units, including two messenger ribonucleic acids (*CRISPR-cas* and *cas3*), one sense ribonucleic acid (*scse2*), and two antisense RNAs (*ascse2-1* and *ascas2-1*) [15].

CRISPR array and *cas* genes are broadly present in the genomes of prokaryotic organisms, such as bacteria and archaea. CRISPR-Cas is a prokaryotic immune system that confers resistance to foreign genetic elements, such as those present within plasmids and phages [16,17,18] that provide a form of acquired immunity. crRNA (CRISPR RNA), which harbors the spacer sequence, helps Cas proteins recognize and cut the DNA of foreign pathogens [19], and RNA-guided Cas proteins can also cleave invading RNA [20], for example, type III-A CRISPR-Cas Csm complex of *Thermus thermophilus* target RNA [21]. Although, it has not been reported in a type I CRISPR-Cas system in vivo, the type I-F CRISPR-Cas system in *Pseudomonas aeruginosa* can cleave the *glpF* mRNA in vitro [22]. CRISPR-Cas components are found in approximately 50% of sequenced bacterial genomes and nearly 90% of sequenced archaea [23]. Besides their role in adaptive immunity, the regulation of gene expression is the most widely reported function of CRISPR-Cas systems, especially the regulation of bacterial virulence and group behavior [24]. Interestingly, the expression of *cas7* (*STY3068*) of a type I-E CRISPR-Cas system has been detected in human macrophages infected by *S*. Typhi [25]. Also, Cas2 of a type II-B CRISPR-Cas system, containing RNA-binding motif, is required for intracellular infection of *Legionella pneumophila* in host cells [26]. Additionally, studies found that *cse1* from this bacterium was expressed in specific growth condition (N-minimal medium) [27], which promoted the expression of the *Salmonella* pathogenicity island 2 genes [28]. Furthermore, the *S*. Typhi CRISPR-Cas locus is regulated by LeuO (LysR-type regulator), H-NS (heat-stable nucleoid-structuring protein), and LRP (leucine-responsive regulatory protein) [27,29], which are involved in pathogenesis [30,31,32,33,34,35]. The *cas* genes of the type I-C CRISPR-Cas system in *Myxococcus xanthus* seem to be strongly embedded within the regulatory circuits that control the fruiting-body formation process and are tightly regulated by various intercellular quorum-sensing (QS) signals and intracellular signaling cascades [36,37]. In addition, some findings support the notion that the CRISPR-Cas system plays a key role in the control of bacterial virulence via the regulation of endogenous genes. Some studies supported that one type II CRISPR-Cas system, which contains a signature *cas9* gene, modulates pathogenesis, thereby controlling bacterial physiology. For example, in the intracellular bacterial pathogen *Francisella novicida*, Cas9 of the type II-B CRISPR-Cas system functions in endogenous bacterial gene *blp* (bacterial lipoprotein) suppression, ultimately promoting both pathogenesis and commensalism [38,39]. Additionally, it is Cas9 of the type II-C CRISPR-Cas system that is essential for attachment and invasion to colorectal epithelial cells by *Campylobacter jejuni* [40]. It is highly likely that type II CRISPR-Cas systems contribute to these virulence traits by regulating endogenous genes. Further, the archaeal type I system encoded by *Pelobacter carbinolicus* has been shown to play a role in the regulation of gene expression [41]. CRISPR spacer #1 of *P. carbinolicus* was shown to match a sequence within *hisS*, the histidyl-tRNA synthetase gene, and interfering with its expression resulted in a mutation and elimination of proteins with multiple closely spaced histidines during evolution. Also, the type I-F CRISPR-Cas system containing a signature *cas3* gene in *Pseudomonas aeruginosa* has been reported to inhibit the production of biofilms by interacting with a specific gene within chromosomally integrated prophages [42,43]. The recent result showed that *Cas3* of *P. aeruginosa* targets the mRNA of the bacterial QS regulator LasR to dampen the recognition of toll-like receptor 4 (TLR4), a type of pathogen recognition receptor (PRR), and thus diminishing the host defense and pro-inflammatory responses in both cells and mouse models [44]. However, it remains unknown whether *Salmonella* CRISPR-Cas systems can regulate endogenous gene expression to impact the virulence of *Salmonella* and evasion from the host immune response. Herein, we uncover a novel pathway employed by *Salmonella* to invade mammalian hosts. The *cas3* deletion strain Δ*cas3* and complementary strain Δ*cas3/pBAD33-CM-cas3* were constructed to study the role of the *cas3* gene in virulence regulation of *Salmonella* by detecting some important virulence phenotypes (e.g., biofilm formation, cell infection) and screening the key virulence genes that may be impacted by *cas3* via RNA-Seq analysis.

In biology, QS signaling confers the ability to detect and respond to the cell population density through complex gene regulation [45]. Bacteria use QS to regulate certain phenotype expressions by self-secreted signaling molecules known as autoinducers (AIs), which in turn coordinate their behaviors. Some QS-regulated phenotypes include biofilm formation, virulence factor expression, and motility while certain traits, such as bioluminescence, nitrogen fixation, and sporulation exist in other bacteria [46]. The modes by which bacteria perceive and transduce AI signals can influence the extent of the QS behavior. There are a variety of recognized AI molecules, including QS molecule autoinducer 2 (AI-2), that are synthesized by LuxS, a key regulator of the QS system [47]. The LuxS/AI-2 QS system has been found in *Salmonella* [48], which regulates the expression of a previously unidentified operon encoding an ATP-binding cassette (ABC)-type transporter, named the *lsr* (*LuxS-regulated*) operon [49]. As the bacterial population density increases, the extracellular concentration of AI-2 also rises until a threshold is reached. AI-2 is transported back into the cell via the Lsr transporter, which is composed of LsrA, LsrB, LsrC, and LsrD, encoded by the *lsr* operon, phosphorylated intracellularly by a kinase, LsrK, and further modified by LsrF and LsrG [50]. In the absence of phosphorylated AI-2 (p-AI-2), LsrR binds the *lsr* promoter to prevent expression of the operon [50,51]. It was found that LsrG is upregulated as part of the *lsr* operon and functions to reduce intracellular phosphorylated AI-2 levels through degradation [52]. This ultimately leads to *lsr* operon repression by reducing intracellular AI-2 levels and allowing LsrR to bind the *lsr* and *lsrRK* promoters. It was found that downregulation of QS by excess LsrR can lower *Salmonella* virulence by hampering *Salmonella* evasion from oxidative killing within macrophages [53]. Also, the QS system may regulate the expression of the *Escherichia coli* type three secretion system 1 (ETT1) encoded by *LEE* operon [54], which is primarily associated with the early stage of infection, where it translocates T3SS (the type three secretion system) effectors across the host cell membrane for bacterial invasion of intestinal epithelial cells, thus triggering intestinal inflammation [55]. We screened *cas3*-related genes annotated as important parts of QS and SPI-1-T3SS systems and elucidated the virulence phenotypes regulated by the system. Furthermore, the study will provide evidence to mechanistically study virulence regulation of CRISPR-Cas systems in *Salmonella*.

## 2. Materials and Methods

### 2.1. Bacterial Strains, Plasmids, Primers, and Growth Conditions

The bacterial strain used in this study was *Salmonella enterica* serovar Enteritidis SE211 containing the *cas3* gene (hereafter *cas3* WT), a poultry isolate, identified and stored at the Laboratory of Quality & Safety Risk Assessment for Livestock and Poultry Products (Wuhan, China), Ministry of Agriculture, China. The suicide plasmid pLP12, expression plasmid pBAD33-CM, and *Escherichia coli* β2163 were supplied by Guangdong KnoGen Biotech Co., Ltd. (Guangzhou, China). *E. coli* DH 5α competence cells were purchased from Beijing TransGen Biotech Co., Ltd. (Beijing, China). The primers used in this study are shown in Table S5. *Salmonella* and *E. coli* strains were grown at 37 °C in Luria-Bertani (LB) broth and LB broth plus 0.3% D-glucose. Antibiotics and additive agent, when necessary, were used at the following concentrations: 20 μg/mL chloramphenicol (CM), 0.3 mM diaminopimelic acid (DAP), and 0.4% L-Arabinose.

### 2.2. Construction of Cas3 Gene Deletion Strain Δcas3

The *cas3* (locus_tag: AV79_16420) gene was amplified by PCR and identified by sequencing. The Δ*cas3* strain was constructed as reported elsewhere [56,57,58]. LB broth medium supplemented with 0.3% D-glucose was used in the process. Briefly, a *cas3* gene fusion fragment was amplified and ligated via PCR, then ligated with pLP12 and subsequently transformed into *E. coli* β2163 grown on LB medium with DAP and CM. The resulting plasmids were introduced into the *cas3* WT strain through conjugation with *E. coli* β2163. After two rounds of selection (first step: CM, second step: L-Arabinose), the mutant carrying the *cas3* gene deletion was validated through PCR using primers (Table S5) corresponding to sequences upstream and downstream of the deletion and subsequent sequencing.

### 2.3. Construction of Cas3 Gene Complementary Strain Δcas3/pBAD3-CM-cas3

Complemented *cas3* mutant strain was constructed according to the previously described method [59]. Briefly, the target gene *cas3* was amplified from the extracted genomic DNA of the *cas3* WT strain. The amplified gene product was subsequently cloned into the pBAD33-CM vector, and then transformed into *E. coli* β2163; the same procedure was applied for plasmid transformation described in the construction of the *cas3* gene deletion strain Δ*cas3* section. Sequencing was performed to confirm the absence of mutation in the target gene. The resulting plasmids were introduced into the *cas3* deletion strain Δ*cas3* and the transformants were selected on LB agar with CM plates. Positive colonies were confirmed by PCR using the targeted gene primers and the primers of the pBAD33-CM plasmid containing the *cas3* gene.

### 2.4. Determination of Standard Growth Curve

The standard growth curves of *cas3* WT, Δ*cas3*, and Δ*cas3/p-cas3* strains were determined by a method described previously [60,61]. An overnight culture of three strains grown at 37 °C was inoculated (1:1000) in fresh LB broth, and incubated at 37 °C in a shaker. Viable colonies (colony-forming units, CFU) of three strains were determined at 0 to 48 h in LB agar after gradient dilution or by detecting the optical density value at 600 nm (OD_600_). These were detected at the following time points: 0.5, 1.5, 2.5, 3.5, 4.5, 5.5, 7.5, 10, 12, 22, 24, and 48 h. The growth curve was generated based on the OD_600_ or the CFU against time, respectively. This experiment was performed in three replicates.

### 2.5. Biofilm Assays

Biofilm formed by *Salmonella* in LB broth was determined and visualized as described previously [62] with some modification. The LB broth uninoculated with a bacteria group was considered as a negative control, and the PAO1 strain, which is one of strong biofilm strains, was used as a positive control [63]. The biofilm formation ability of the *cas3* WT, Δ*cas3*, and Δ*cas3/p-cas3* strains in the static condition was examined visually after growth in 4 mL of LB broth at 28 °C for 96 h, and then the biofilms were stained by 1% crystal violet, washed by distilled water, and dissolved by 30% glacial acetic acid. Absorbance at 590 nm was measured with an automated microplate reader (BioTek, Winooski, VT, USA), and the biofilm index was defined by the optical density value.

Crystal violet quantification of the biofilms formed by the *S*. Enteritidis strains was performed using a microtiter plate assay as described previously [64,65]. An overnight culture of bacterial strains in LB was diluted 1:10 and used to inoculate 96-well polystyrene microtiter plate (Corning, New York, USA) wells containing 90 μL of LB, and the plate was incubated at 37 °C for 48 h. The LB was discarded and the wells were gently washed with phosphate-buffered saline (PBS), to which 100 μL of 1% crystal violet was then added. The biofilms were stained for 20 min and resolved by the addition of 100 μL of ethanol after the wells were gently washed with distilled water. The absorbance at 590 nm was measured with an automated microplate reader, and the biofilm index was defined by the OD value. Assays were repeated three times with three technical replicates and the results were averaged. Meanwhile, the LB broth uninoculated with bacteria was considered as a negative control, and 2 × OD_NTC_ was considered as the reference biofilm formation.

### 2.6. Cell Culture Infection Assays

Cells grown to a cell density of ~2 × 10^5^ cells/well in 24-well cell culture plates (Corning Cell-Bind) were infected with *Salmonella* strains at multiplicities of infection (MOI) of 100:1 bacteria:cell ratio. Invasion and intracellular survivability were accessed by the colony-forming units (CFU) count assay as previously described [66]. For MH-S, RAW264.7, SW480, SW620, and IPEC-J2 cells, the bacterial numbers of internalization were assessed at the post-infection time points indicated in the figures, respectively. After that, monolayers were washed (three times) using DMEM (Dulbecco modified eagle medium) without antibiotic to get rid of the extracellular unbound bacteria, followed by a change of the cell culture medium containing 100 μg/mL gentamycin, and further incubation for 90 min to kill extracellular bacteria. Next, cell culture medium was removed from duplicate wells of infected cells, and the cells were washed twice with PBS, and treated with freshly prepared sterile 0.3% Triton X-100 in PBS. Finally, intracellular bacteria were released, and the bacterial CFUs were determined by plating samples to Bismuth Sulfite (BS) agar dishes. Also, 4 h post-infection, MH-S cells and intracellular bacteria were observed by laser scanning fluorescence confocal microscope (LSCM) under an LSM 510 Meta Confocal Microscope (Carl Zeiss Micro Imaging, Thornwood, NY, USA) after staining with SYTO^®^ green-fluorescent nucleic acid stains (Invitrogen, Carlsbad, CA, USA). In addition, the cell death of MH-S 2 h post-infection was measured by propidium iodide (PI) uptake and flow cytometry as the protocol described [67]. Additionally, a positive control with dead cells and an unstained control were performed. The results were based on the Flow Jo V10. Cells were gated based on FSC and SSC using a linear scale to gate out cellular debris. The fluorescence intensity was then determined for PI and plotted using a linear scale. The percentage of dead cells was analyzed.

### 2.7. Determination of the 50% Lethal Dose (LD_50_) for SPF Chicken during Salmonella Oral Infection

The one-day specific pathogen-free (SPF) chickens (10 animals per group) were orally infected by a stomach tube by logarithmically grown doses: About 10^8^, 10^9^, 10^10^, and 10^11^ CFU/chicken of the *Salmonella* strain. Bacteria were dissolved in 1 mL of 0.9% NaCl. The chickens were observed daily for 8 days. The lethal dose was estimated using the Karber method: LD_50_ = lg^−1^ [X_k_-d (∑P_i_ – 0.5)], SD = $\frac{{\mathrm{lg}}^{-1}({\mathrm{lgLD}}_{50}\;+\;1.96\;\times \;i\sqrt{\frac{\sum \mathrm{Pi}\;-\;\sum {\mathrm{Pi}}^{2}}{n\;-\;1})}\;-{\;\mathrm{lg}}^{-1}({\mathrm{lgLD}}_{50}\;-\;1.96\;\times \;i\sqrt{\frac{\sum \mathrm{Pi}\;-\;\sum {\mathrm{Pi}}^{2}}{n\;-\;1})}}{2}$. In these formulas, X_k_ represents log_10_ (maximum dose), d represents lg (class interval), P represents the mortality of each dose group, i represents each group, and n represents the number of animals per group.

### 2.8. Transcriptome Analysis by RNA-Seq

After the phenotypic determination of *cas3* WT and Δ*cas3* strains, RNA-Seq was performed as described previously [68]. The *cas3* WT and Δ*cas3* strains (three for each sample) were harvested at log phase. 

#### 2.8.1. RNA Isolation, Library Construction, Sequencing, and Sequence Data Filtering

By following the manufacturer’s instructions, total RNAs were isolated from the samples using a RNAprep pure Cell/Bacteria Kit following the manufacturer’s instructions (Tiangen Biotech CO., LTD., Beijing, China), and then treated with RNase-free DNase I (Ambion Inc., Austin, TX, USA) to remove DNA. The purity and integrity of total RNA was checked with an Agilent 2100 system with RIN (RNA Integrity Number) over 7. The rRNA was removed with a Ribozero kit and paired-end RNA-Seq was performed on the Illumina HiSeq platform according to the manufacturer’s protocol (paired-end sequencing; 100 bp fragment) at Shanghai Personalgene Biotechnology. Briefly, synthesis of first-strand cDNA was carried out using the Super-ScriptII (Invitrogen, Carlsbad, CA, USA) in the presence of random hexamer primers. The synthesis of second-strand cDNA was performed before end repair and addition of deoxyadenine. Ligation of the DNA fragment was performed by using a truSeq adapter and amplified with truSeq PCR primers for sequencing. We then removed the reads with the adaptor by Cutadapt software (Version 1.2.1) and the low-quality reads. Three independent experiments of each group were performed and sequenced.

#### 2.8.2. Differentially Gene Expression (DEGs), Clustering Analysis, and Functional Analysis of DEGs

The resulting sequences were then aligned to the reference genome of strain EC20120051 (GenBank Accession: NZ_CP007433.2) in order to create a transcriptome map using Bowtie2 (http://bowtie-bio.sourceforge.net/index.shtml). Gene quantification was calculated by the FPKM (fragments per kilobase per million fragments) method [69]. When FPKM > 1, it is considered that the gene is expressed. The DESeq analysis between the *cas3* WT and Δ*cas3* bacterial samples was conducted using the DESeq package of R. *p*-values used to identify the significance of DEGs were estimated according to the hypergeometric test. The genes with a *p*-value ≤0.05 and |log2fold change (Δ*cas3*/*cas3* WT)| ≥ 1 were identified as DEGs. The clustering analysis and data visualization of DEGs was performed by the heatmap package of R. The functional annotations of DEGs were carried out by gene ontology (GO) from an internationally established system (GO; http://www.geneontology.org). This system comprehensively describes the genes’ properties and provides their products in any organism. The corrected *p*-value of GO terms (*p* < 0.05) was considered significantly enriched by DEGs. DEGs analyses were performed on the basis of the biological process, molecular function, and cellular components. The Kyoto Encyclopedia of Genes and Genomes (KEGG) database was used to determine the molecular pathways for the DEGs. 

#### 2.8.3. Data Validation by RT-qPCR

The RNA sequencing results were verified by RT-qPCR (Bio-Rad CFX 96™, Hercules, CA, USA). For that, 26 genes were selected based on their function classification and differential expression in the RNA-Seq results. These included the *cas* operon, *lsr* operon, and T3SS genes. For RT-qPCR, briefly, one microgram of total RNA was reverse transcribed into complementary DNA (cDNA) using the HiScript II Q Select RT SuperMix for qPCR (+gDNA wiper) (Vazyme, Nanjing, China). The cDNA was amplified by RT-qPCR using SYBR Green Real time PCR Master Mix (Takara, Japan). Relative quantification of gene expression was calculated using the 2^−ΔΔCt^ method and normalized to reference gene 16S rRNA in each sample, in which *cas3* WT was used as a control. The primers used in RT-qPCR are listed in the Table S5.

### 2.9. Statistical Analysis

For statistical analyses, GraphPad Prism 7 software (San Diego, CA, USA) was used to determine the mean ± standard deviation (MSD) and significance level by applying a one-way ANOVA with Dunnet’s multiple comparison tests comparing *cas3* WT, Δ*cas3*, and Δ*cas3/p-cas3* for in vitro experiments, such as biofilm formation, invasion, and intracellular survivability. In the figures, one asterisk (*) and two asterisks (**) represent statistically significant differences at *p* ≤ 0.05 and *p* ≤ 0.01, respectively, in the comparison of the Δ*cas3* and Δ*cas3/p-cas3* strains to the *cas3* WT strain. A Pearson’s correlation coefficient (r) was computed between the RNA-Seq and RT-qPCR results, with a significance cutoff of *p* ≤ 0.05.

## 3. Results

### 3.1. Deletion of Cas3 has No Effect on Bacterial Growth

**Figure 2:**
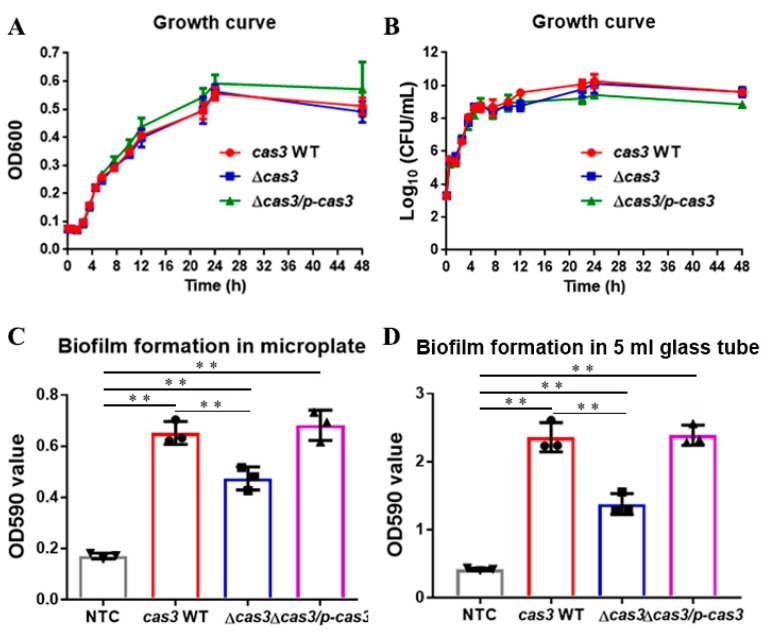
Impact of *cas3* on the growth and biofilm formation of *Salmonella*. (**A**,**B**) Deletion of *cas3* has no effect on bacterial growth. (**C**) Biofilm formation of *cas3* WT, Δ*cas3*, and Δ*cas3/p-cas3* after 48 h of incubation at 37 °C under static conditions quantified by crystal violet. (**D**) Biofilm formation in LB (Luria-Bertani) broth was detected and quantified by crystal violet after 96 h of incubation at 28 °C. NTC, non-treated control; Bars, mean ± SD; n = 3; ** *p* ≤ 0.01; * *p* ≤ 0.05 (one-way ANOVA plus Dunnet’s multiple comparisons test).

The *Salmonella* WT strain *cas3* WT, *cas3* deletion strain Δ*cas3*, and complementary strain Δ*cas3/pBAD33-CM-cas3* were grown separately in LB broth for the determination of the standard growth curve. Compared to the growth of *cas3* WT, Δ*cas3*, and Δ*cas3/p-cas3* strains under shaking conditions (Figure 2A,B), we found that there were no significant differences in the growth among the *cas3* WT, Δ*cas3*, and Δ*cas3/p-cas3* strains.

### 3.2. Cas3 Deletion Impacts Bacterial Biofilm Formation and Cell Infection

We determined the biofilm of the *cas3* WT, Δ*cas3*, and Δ*cas3/p-cas3* strains in vitro under static conditions. Crystal violet quantification analysis of the biofilms showed that all three strains could form a biofilm in the LB broth after incubation for 48 h at 37 °C. However, it was confirmed that the biofilm-forming ability of the *cas3* WT was significantly stronger than that of Δ*cas3* while the complementation of the *cas3* gene (Δ*cas3/p-cas3* strain) restored the WT phenotype (Figure 2C). 

In addition, the biofilm phenotypes of the three strains were visualized as a floating pellicle and a ring of bacteria adhered to the tube wall at the air–liquid interface in the LB broth after incubation for 96 h at 28 °C (Figure S1A). Then, the biofilm was stained by crystal violet (Figure S1B,C). The biofilm formation phenotypes of the three strains were the same as that of the strong biofilm strain *Pseudomonas aeruginosa* PAO1, positive control for the biofilm (Figure S1D), but a significant decrease in biofilms was observed in the Δ*cas3* strain compared to the *cas3* WT and Δ*cas3/p-cas3* strains. Similarly, by crystal violet quantification, the biofilms of the *cas3* WT and Δ*cas3/p-cas3* strains were significantly stronger than those of the Δ*cas3* strain (Figure 2D). 

**Figure 3:**
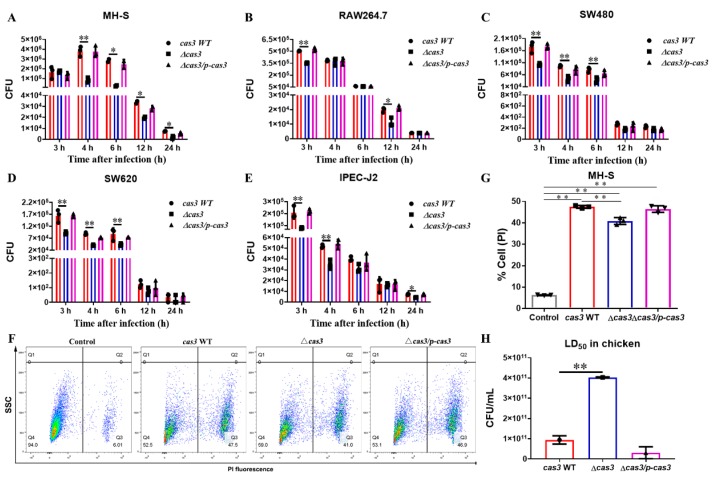
Deficiency of *cas3* in *Salmonella* weakens the bacterial virulence to host cells. (**A**–**E**) *cas3* deletion decreased the invasion of *Salmonella* to different cells and the *cas3* WT and Δ*cas3/p-cas3* strain showed a higher count of intracellular bacteria at different time points compared to the Δ*cas3* strain. This figure shows the CFU (Colony-Forming Unit) of each strain in the cells at different times after infection and the results were based on the CFU assays obtained from three independent experiments. (**F**,**G**) Measurement of murine alveolar macrophage cell MH-S death by propidium iodide (PI) uptake. (**F**) The sample of uninfected MH-S cells was regarded as a control. The MH-S cells of trial groups were infected by *cas3* WT, Δ*cas3*, and Δ*cas3/p-cas3* strains, respectively, for 2 h and assayed by PI uptake. The percentage of dead cells (Q3 region) is shown, respectively, and the statistical results of the three strains are shown as a column diagram (**G**). (**H**) LD_50_ (50% lethal dose) of *Salmonella* for chickens following oral infection. Bars, mean ± SD; n = 3; ** *p* ≤ 0.01; * *p* ≤ 0.05 (one-way ANOVA plus Dunnet’s multiple comparison test).

We observed that the *Salmonella* Δ*cas3* strain exhibited lower invasion to mouse macrophage cells MH-S and RAW264.7, human colon cancer cells SW480 and SW620, and porcine small intestinal epithelial cells IPEC-J2 than the *cas3* WT and Δ*cas3/p-cas3* strain (Figure 3A–E). At 4 h post-infection, a significant decrease in invasion to macrophage cell MH-S was observed in the Δ*cas3* strain compared to the *cas3* WT strain, and the apparent change was eliminated in the Δ*cas3/p-cas3* strain. Then, at 24 h post-infection, the intracellular survival of the Δ*cas3* strain remained significantly lower than both the *cas3* WT and Δ*cas3/p-cas3* strains. RAW264.7 cells infected with *Salmonella* Δ*cas3* showed decreased bacterial intracellular survival at the time points of 3 and 12 h after infection. Similarly, decreased intracellular bacteria survival was observed while epithelial cells were infected after different times by the Δ*cas3* strain than the *cas3* WT strain or complementary strain Δ*cas3/p-cas3*. 

Laser scanning confocal microscopy (LSCM) showed significant internalization of the *cas3* WT and complemented strains in MH-S cells stained with SYTO 9, one of the green-fluorescent nucleic acid stains, compared to the *cas3* deletion strain (Figure S1E). These results showed that the invasion of *Salmonella* into cells and the intracellular survival ability decreased after *cas3* gene deletion mutant infection. Next, we examined the host cells’ survival and found that the survival of MH-S cells was better in the Δ*cas3* strain group compared to both the *cas3* WT and Δ*cas3/p-cas3* strain groups (Figure 3F). Quantification of the flow cytometry results showed that the percentage of dead cells infected by the Δ*cas3* strain was markedly decreased (Figure 3G; a positive control and an unstained control are shown in Figure S1F), suggesting that CRISPR-Cas deficiency may be associated with a decrease in bacterial virulence.

### 3.3. Deletion of Cas3 Increased the LD_50_ Dose for SPF Chickens during Oral Infection

Mortality rates were used for the calculation of the LD_50_ lethal dose, which for the SPF chicken model was infected with 9.378 ± 2.075 × 10^10^ CFU/animal for the *cas3* WT, 3.07 ± 2.944 × 10^10^ CFU/animal for the Δ*cas3/p-cas3*, and 4.029 ± 0.028 × 10^11^ CFU/animal for the Δ*cas3* strain, respectively (Table S1). The LD_50_ concentration of the Δ*cas3* strain was significantly increased compared to the *cas3* WT strain, and there was no significant difference between the Δ*cas3/p-cas3* and *cas3* WT strains (Figure 3H).

Chickens that died during the experiment were analyzed postmortem, and *Salmonella* was detected in the organs (culture from the blood, liver, spleen, and caecum).

### 3.4. Transcriptomics Analysis Reveals Differentially Expressed Genes between Cas3 WT and Δcas3 Strains 

To determine the different regulators involved in virulence, we investigated the gene expression profile of the Δ*cas3* and *cas3* WT strains. The results revealed that 141 genes were differentially expressed, of which 60 genes were upregulated and 81 genes were downregulated, in the Δ*cas3* strain. The gene expression levels are shown in an MA plot (Figure S2A) and volcano plot (Figure S2B). Interestingly, all the genes in the *cas* operon except *cas3* were upregulated (Table 1). By analyzing these differentially expressed genes (DEGs), we found that in the most significant DEGs, several important genes encoded by the *lsr* operon were strongly upregulated, which is related to the QS system. Also, the expression of some biofilm-related genes related to fimbriae was changed (Table 2) [49,50,52]. In addition, another important group of DEGs encoded by SPI-1 appeared to be related to T3SS, a key regulator of *Salmonella* virulence to host cells (Table S2). Finally, cluster analysis of all DEGs was performed, and the results are shown in Figure S2C. 

#### 3.4.1. GO and KEGG Analysis Identifies Functional Relevance to DEGs

**Figure 4:**
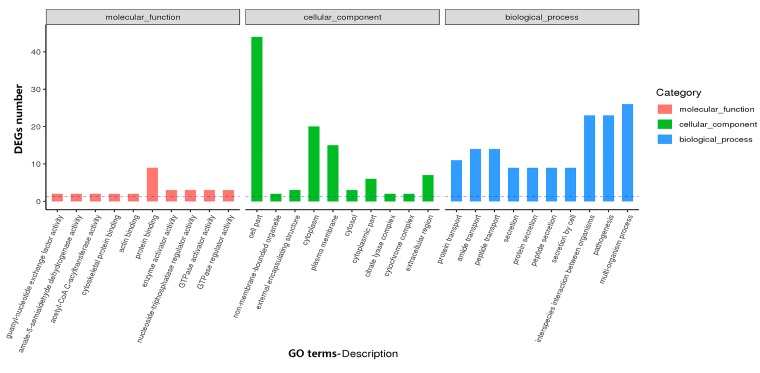
Gene ontology (GO) annotations of differentially expressed genes (DEGs) and classification graph of GO terms for DEG. GO includes the molecular function (MF), cellular component (CC), and biological process (BP).

The DEGs between the Δ*cas3* and *cas3* WT strains were assessed according to the GO classification to investigate the potential biological functions in which the DEGs might be involved in. Based on the GO annotation, the top three categories of the DEGs were “cell part” (CC, 44 genes), “multi-organism process” (BP, 26 genes), and “pathogenesis” (BP, 23 genes) (Figure 4). All the significantly enriched GO terms are presented in Table S3.

Then, DEGs were mapped to the KEGG database and linked to important pathways based on the entire transcriptome background (Table S4). It showed that the top three frequent categories of DEGs mapped pathways were “environmental information”, “human diseases”, and “metabolism” (Figure S3A). In detail, the 20 pathways that were most significantly enriched are shown in Figure S3B, in which the top two significantly enriched pathways were “bacterial invasion of epithelial cells” (FDR = 1.15792 × 10^−5^) and “*Salmonella* infection” (FDR = 0.004114819).

#### 3.4.2. The Patterns of DEGs are Similarly Revealed by RT-qPCR Analysis

**Figure 5:**
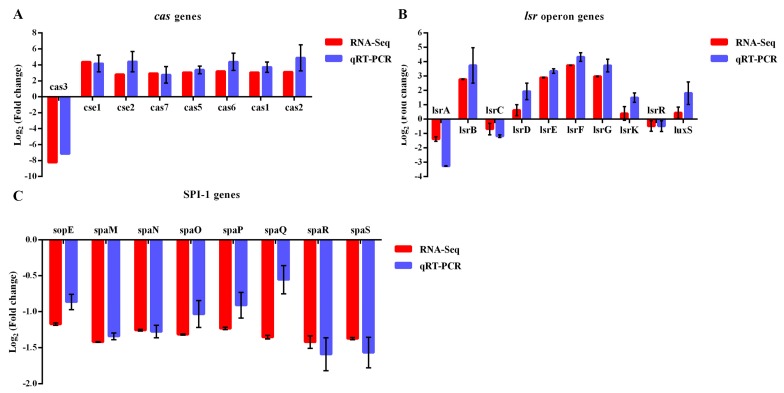
Comparison of the expression level of some key genes acquired from RNA-Seq and RT-qPCR, including *cas* operon genes (**A**), *lsr* (*LuxS-regulated*) operon genes (**B**), and some of the SPI-1-T3SS genes (*Salmonella* pathogenicity island 1 genes related to the type three secretion system) (**C**).

As shown in Figure 5A–C, the expression of the selected genes (*cas* operon, *lsr* operon, and SPI-1 genes) from the RNA-Seq data was consistent with the results revealed by the RT-qPCR analysis, suggesting that the RNA-seq results were robust and reliable. 

## 4. Discussion

*Salmonella* is one of the most common and important microbial pathogens; however, limited knowledge is available regarding the distinct pathogenesis mechanisms associated with *Salmonella* infections of hosts [70]. This study reports that Cas3 nuclease of type I-E CRISPR-Cas systems in *Salmonella* SE211 (*cas3* WT strain) has an unconventional function in altering its own virulence by regulating the *lsr* operon, enabling stronger biofilm formation, invasion to host cells, and intracellular survivability, and leading to a decrease of live cells or chickens infected by *Salmonella*. The endogenous targeting by CRISPR-Cas systems may boost bacterial virulence. Bacterial endogenous mRNA or invading RNA targeting has been reported in type II and III CRISPR-Cas systems [21,26,38], as well as in type I-F [44]. However, targeting a type I-E CRISPR-Cas system has not been investigated to date.

It has been well recognized that biofilm formation helps *Salmonella* to survive under adverse environment [71]. Importantly, biofilm formation is one of the important virulence determinants of *Salmonella* and is also associated with resistance against the response of the host immune system [72,73,74]. A previous study showed that biofilm formation was decreased in the *Streptococcus* mutants *cas3* deficiency strain, encoding a type I-C CRISPR-Cas system [75]. In this study, we firstly found that the type I-E CRISPR-*cas3* WT strain of *Salmonella* has a stronger ability to form biofilms than the Δ*cas3* strain. These results suggest that the *cas3* gene may participate in the regulation of bacterial biofilm formation. A study in *C. jejuni*, containing a type II-C CRISPR-Cas system, also supports our findings that the *C. jejuni* WT strain has a stronger capability to form biofilms than mutant strains [66]. This showed that CRISPR-Cas systems play a role in biofilm formation to alter virulence.

CRISPR-Cas systems are often associated with bacterial pathogenicity, especially their invasion to eukaryotic cells [76]. The invasion of *Salmonella* to host cells is considered as the main feature in its pathogenesis [77]. Several studies discovered that *Salmonella* has the ability to reproduce and survive once it entered the host cells [78]. In the present study, bacterial invasion and intracellular survival ability of *cas3* WT and Δ*cas3* were detected to determine their virulence. Our results showed that the counts of Δ*cas3*, including invasion and survival in host cells, were significantly decreased compared to its WT strain and complementary strain. It was demonstrated that the invasion and intracellular survival of *Salmonella* containing a type I-E CRISPR-Cas system are greater than those with CRISPR-Cas deficiency. Furthermore, the mortality of the host eukaryotic cells after Δ*cas3* strain infection was significantly decreased, indicating that *cas3* of *Salmonella* is likely beneficial for bacterial infection. Importantly, using an established chicken infection model, we demonstrated that the virulence of the *cas3* deficiency strain was lower (the LD_50_ was higher) compared to the wide type strain, and the virulence of the *cas3* complementary strain was completely restored, which suggests that the *cas3*-harboring strain may result in a high mortality in chickens. In a previous study regarding with the virulence of *Enterococcus faecalis* isolates, the type II CRISPR1-Cas-harboring strain induced a higher mortality in mice when equal inoculum of both strains was used [79], which is consistent with our study.

To explore the underlying mechanism of the *cas3*-induced highly pathogenicity, we performed transcriptome sequencing, which offers an efficient method for profiling global gene expression patterns, to illuminate the role of Cas3 protein in modulating the expression of endogenous genes. Upon the transcriptome analysis of *cas3* WT and Δ*cas3* strains, we observed that the majority of genes related to bacterial virulence were downregulated in the Δ*cas3* strain (Table S2). 

Most DEGs were located at *Salmonella* pathogenicity island 1 (SPI-1), which encodes several effector proteins that may trigger the invasion and internalization of bacteria into epithelial cells by mediating actin cytoskeletal rearrangements. These effectors are translocated into the host cells by means of T3SS that is also encoded within SPI-1, on which *Salmonella* invasion is dependent [77]. T3SS directly injects bacterial effectors into the cytosol of host cells to initiate cytoskeletal rearrangement and alter signal transduction process [80]. The *prg* (*KJIH*)/*org* (*ABC*) and *inv* (*HFGEA*)/*spa* (*KMNOPQRS*) operons encode the needle complex per se, and the *sip* (*BCDA*) operon encodes the effector proteins and translocon, a pore-forming structure that embeds in the host cell membrane and delivers these effectors into the cytosol of host cells [77]. In addition, several chaperones are also encoded within SPI-1. Through specific binding to their targets (secreted or effector proteins), these chaperones protect SPI-1-related proteins from degradation, prevent premature interactions, and/or mediate their recognition by T3SS. For example, effector protein SptP is related to the disruption of the actin cytoskeleton by antagonizing SopE (*Salmonella* outer protein E), SopE2, and SigD (*Salmonella* invasion gene) [81]. In the current study, the expression of all of these genes was significantly decreased, so the SPI-1-T3SS operon may play an important role in Cas3-regulated *Salmonella* infection, especially the invasion process. This might be one reason why we found less virulence in the Δ*cas3* strain as compared to the *cas3* WT strain. Furthermore, it is of importance to determine how the CRISPR-Cas system regulates SPI-1-T3SS expression in *Salmonella*. 

In addition, some DEGs were implied as fimbria or curli genes influencing biofilm formation, including *safA*, *safB, safC*, and *safD* (downregulated), and *bdm* (upregulated). Previous research showed that deletion of *saf* in *Salmonella* decreased biofilm production to the level of about 85% of the wild-type strain [82]. Additionally, biofilm-dependent modulation protein (*bdm*), encoding a putative 71-amino acid protein, could act as a transcriptional activator for genes that are involved in flagella formation and was shown to be downregulated in biofilms [83]. It is noteworthy that deficiency of the *cas3* gene changed the expression of some genes involved in QS. The QS system is a phenomenon in which pathogenic bacteria produce signaling molecules that are involved in cell-to-cell communication, the production of virulence factors, biofilm maturation, and several other functions [84]. Many gram-positive bacteria utilize peptide QS systems to control gene expression. For example, the QS system in *Staphylococcus aureus* is thought to impact virulence by affecting the biofilm formation. *P. aeruginosa* is a gram-negative bacterium and serves as a model to study bacterial QS systems. It was found that QS signaling regulates the production of several extracellular virulence factors, promotes biofilm maturation [85,86], tissue destruction, and death [87], and also affects the interaction of bacteria with host cells and the host immune response [87,88]. This suggests that QS signaling plays a key role in the pathogenesis of *P. aeruginosa*. Another bacterial species that uses QS to control virulence gene expression is *Escherichia coli*, with which the *Salmonella* genome encodes a similar QS system, LuxS/AI-2. Additionally, the genetic studies in enterohaemorrhagic *E. coli* (EHEC) and enteropathogenic *E. coli* revealed that LuxS, a key regulator of the QS system, controls the expression of the T3SS system encoded by the locus of enterocyte effacement (LEE) pathogenicity island [54], but no mechanistic links has been established between QS and T3SS systems. Additionally, transcriptomic studies have revealed that LuxS, is a global regulator in EHEC, and can control the expression of over 400 genes [89]. Most of these genes have functions related to bacterial virulence, such as flagellar motility, surface adhesion, and Shiga toxin production [90]. In the present study, we found four *lsr* genes, such as *lsrF*, *lsrG*, *lsrB*, and *lsrE* with significantly upregulated expression in the Δ*cas3* strain, especially the *lsrF*. LsrF and LsrG process p-AI-2 further to form a product that is unable to interact with LsrR in *S*. Typhimurium [50,91]. A study showed that LsrB can decrease the transcription of the *lsr* operon in response to AI-2, and the LsrB mutant strain has a delayed response for transporting AI-2 entering the cells [50]. So far, the function of LsrE is unclear, although sequence analysis suggests that *lsrE* is homologous to *rpe*, which encodes the ribulose phosphate epimerase. In the *cas3* WT strain containing a CRISPR-Cas system, *lsrF* and *lsrG* are lowly expressed, which mayresult in the accumulation of p-AI-2. This could induce transcription of the lsr operon by inactivation of the LsrR repressor. Also, the low-level expression of *lsrB* may promote *lsr* operon expression. Furthermore, it was previously found that the deficiency of *cas3* in the type I-F CRISPR-Cas system of *P. aeruginosa* increased the mRNA expression of the bacterial QS regulator LasR to promote recognition of host cells by TLR-4, thus regulating the virulence to both host cells and mice in vivo [44]. More recently, we delineated that type I CRISPR-Cas may regulate the host’s inflammasome activation by altering mitochondrial DNA release and subsequent autophagy [92]. From a different angle, QS systems can also potently upregulate the gene expression of CRISPR-Cas [93,94]. Furthermore, sRNA may also regulate the crRNA biogenesis of CRISPR-Cas by inhibiting transcription termination [95]. In our current study, upregulated expression of QS system genes was also observed but is a different one in *Nature*. In addition, most of the downregulated DEGs were encoded as SPI-1-T3SS system proteins, which mainly regulate the bacterial invasion of epithelial cells. 

**Figure 6:**
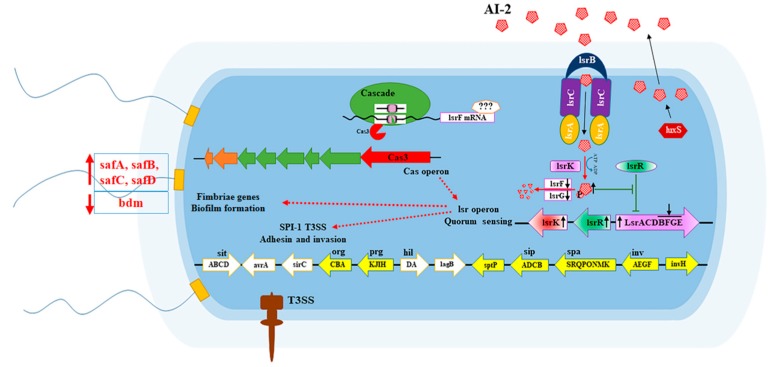
Proposed mechanisms of the CRISPR-Cas system in regulating biofilm-forming-related genes and SPI-1 (*Salmonella* pathogenicity island 1) expression by degrading *lsrF* encoded by *lsr* operon. Cas3 may target and downregulate the expression of *lsrF*, and then delay the degradation of p-AI-2. Accordingly, activated AI-2 is increased, which then inhibits the activity of LsrR protein to increase the expression of the *lsr* operon. However, the expression of *lsrF* remains inhibited by this time. Finally, the expression of the biofilm-forming-related genes and SPI-1-T3SS genes are increased, and the biofilm formation and invasion of *Salmonella* to epithelial cells are activated.

The above results suggest that *cas3* plays a role in the regulation of QS genes, which are essential for the biofilm formation and invasion of bacteria. Mechanistically, Cas3 may target and downregulate the expression of *lsrFGBE*, and then delay the degradation of p-AI-2. Resultantly, the activated form of AI-2 is increased that results in the inhibition of the activity of LsrR protein and the upregulation of the *lsr* operon, but the expression of *lsrFGBE* remains to be inhibited. After that, the biofilm-forming-related genes and SPI-1-T3SS genes are promoted, and the virulence, especially the biofilm formation ability and the invasion of *Salmonella* to epithelial cells, is activated (Figure 6).

## 5. Conclusions

In summary, this study revealed that the *cas3* gene participates in the regulation of biofilm formation in *Salmonella*. Importantly, deletion of the *cas3* gene made *Salmonella* less pathogenic to host cells and chickens. Accordingly, regulatory compounds could be designed and synthesized to block the expression of the *cas3* gene to decrease the virulence of bacteria. In addition, this knowledge provides a new way for understanding the functions of CRISPR-Cas systems in biology and pathogenicity of *Salmonella*. Our findings also provide important information for the correlation study of CRISPR-Cas systems and bacterial virulence. Furthermore, it’s also necessary to explore the molecular mechanism by which *cas3* and other CRISPR components impact the virulence in *Salmonella*.

## Figures and Tables

**Table 1:** *Cas* operon expression in *Salmonella* Δ*cas3* (*cas3* deficiency strain) versus *cas3* wild type strain.

Locus Tag	Gene	Protein	Fold Change (Δ*cas3*/*cas3* WT)	*p*-Value
AV79_RS14235	*cas3*	type I CRISPR-associated protein DNA helicase Cas3	−301.27	5.331 × 10^−11^
AV79_RS14230	*cse1*	type I-E CRISPR-associated protein Cse1	20.47	2.96 × 10^−5^
AV79_RS14210	*cas6*	type I-E CRISPR-associated protein Cas6	9.11	6.002 × 10^−5^
AV79_RS14200	*cas2*	CRISPR-associated protein Cas2	8.66	0.0003026
AV79_RS14215	*cas5*	type I-E CRISPR-associated protein Cas5	8.34	0.0002544
AV79_RS14205	*cas1*	CRISPR-associated protein Cas1	8.25	0.0001503
AV79_RS14220	*cas7*	type I-E CRISPR-associated protein Cas7	7.68	0.0004107
AV79_RS14225	*cse2*	type I-E CRISPR-associated protein Cse2	6.98	0.0006198

**Table 2:** Expression of the quorum-sensing (QS) system and fimbrial genes in the *Salmonella* Δ*cas3* strain versus *cas3* WT strain.

Gene	Protein	Fold Change (Δ*cas3*/*cas3* WT)	*p*-Value	Function
*lsrF*	putative aldolase, 3-hydroxy-5-phosphonooxypentane-2, 4-dione thiolase LsrF	13.48	0.01	Involved in the degradation of phospho-AI-2, thereby terminating induction of the lsr operon and closing the AI-2 signaling cycle. Catalyzes the transfer of an acetyl moiety from 3-hydroxy-5-phosphonooxypentane-2, 4-dione to CoA to form glycerone phosphate and acetyl-CoA.
*lsrG*	(4S)-4-hydroxy-5-phosphonooxypentane-2, 3-dione isomerase, autoinducer-2 (AI-2) modifying protein LsrG	7.90	0.02	Involved in the degradation of phospho-AI-2, thereby terminating induction of the lsr operon and closing the AI-2 signaling cycle. Catalyzes the conversion of (4S)-4-hydroxy-5-phosphonooxypentane-2, 3-dione (P-DPD) to 3-hydroxy-5-phosphonooxypentane-2, 4-dione (P-HPD).
*lsrE*	Ribulose-phosphate 3-epimerase, Putative epimerase LsrE	7.47	0.03	Cofactor.
*lsrB*	Autoinducer 2-binding protein LsrB	6.88	0.03	Part of the ABC transporter complex LsrABCD involved in autoinducer 2 (AI-2) import. Binds AI-2 and delivers it to the LsrC and LsrD permeases.
*lsrA*	putative ABC transporter ATP-binding protein, Autoinducer 2 import ATP-binding protein LsrA	−2.60	0.15	Part of the ABC transporter complex LsrABCD involved in autoinducer 2 (AI-2) import. Responsible for energy coupling to the transport system.
*lsrR*	transcriptional repressor LsrR	−1.39	0.36	In the absence of autoinducer 2 (AI-2), represses transcription of the lsrACDBFGE operon and its own transcription. In the presence of AI-2, LsrR is inactivated by binding phospho-AI-2, leading to the transcription of the lsr genes
*lsrD*	ABC transporter membrane protein, Autoinducer 2 import system permease protein LsrD	1.55	0.38	Part of the ABC transporter complex LsrABCD involved in autoinducer 2 (AI-2) import. Probably responsible for the translocation of the substrate across the membrane.
*lsrC*	sugar transport protein, Autoinducer 2 import system permease protein LsrC	−1.61	0.40	Part of the ABC transporter complex LsrABCD involved in autoinducer 2 (AI-2) import. Probably responsible for the translocation of the substrate across the membrane.
*lsrK*	autoinducer-2 (AI-2) kinase	1.32	0.48	Catalyzes the phosphorylation of autoinducer-2 (AI-2) to phospho-AI-2, which subsequently inactivates the transcriptional regulator LsrR and leads to the transcription of the *lsr* operon. Phosphorylates the ring-open form of (S)-4, 5-dihydroxypentane-2, 3-dione (DPD), which is the precursor to all AI-2 signaling molecules, at the C5 position.
*luxS*	S-ribosylhomocysteinase	1.36	0.40	Involved in the synthesis of autoinducer 2 (AI-2) which is secreted by bacteria and is used to communicate both the cell density and the metabolic potential of the environment. The regulation of gene expression in response to changes in cell density is called quorum sensing. Catalyzes the transformation of S-ribosylhomocysteine (RHC) to homocysteine (HC) and 4, 5-dihydroxy-2, 3-pentadione (DPD).
*safA*	Lipoprotein, Saf-pilin pilus formation protein safA	−3.79	0.02	One of major fimbrial subunits.
*safB*	pili assembly chaperone protein SafB	−3.28	0.02	Involved in the organization of pilus and the chaperone-mediated protein folding.
*safC*	atypical fimbria outer membrane usher SafC	−2.30	0.02	Involved in pilus assembly and positive to fimbrial usher porin activity.
*safD*	fimbrial structural subunit SafD	−2.72	0.01	Part of fimbrial structures.
*bdm*	biofilm-dependent modulation protein BDM	3.49	0.04	Bdm acts as a transcriptional activator for genes that are involved in the flagella formation and was shown to be downregulated in biofilms.

## Supplementary Materials

The following are available online at https://www.mdpi.com/2076-0817/9/1/53/s1, Figure S1: Impact of *cas*3 on biofilm formation and cell infection of Salmonella, Figure S2: Differentially expressed genes (DEGs) between Δ*cas*3 and *cas*3 WT strains, Figure S3: Kyoto Encyclopedia of Genes and Genomes (KEGG) pathway classification and enrichment of DEGs, Table S1: Mortality data for chickens following oral infection by Salmonella, Table S2: The differentially expressed genes (DEGs) located at SPI-1 and related to SPI-1-T3SS in Salmonella Δ*cas*3 strain versus *cas*3 WT strain, Table S3: The significantly enriched GO terms of DEGs, Table S4: The KEGG pathways map of DEGs, Table S5: Primers used in this study.
