# Supplementary material for: CRISPR-cas3 of Salmonella Upregulates Bacterial Biofilm Formation and Virulence to Host Cells by Targeting Quorum-Sensing Systems
Source: Pathogens. 2020 Jan 10;9(1):53. doi: 10.3390/pathogens9010053 (PMC7168661; doi:10.3390/pathogens9010053)
Supplement: Supplementary file 1 [file pathogens-09-00053-s001.zip › Table S5 primers.docx]

Table S5. Primers used in this study

| Primer name | Nucleotide sequence (5’ to 3’) | Purpose of amplification | Size/bp |
| --- | --- | --- | --- |
| Cas3-MF1 | ggaatctagaccttgagtcgAGCCCGGTACGATAGCCTTTG | *cas3* upstream flanking sequences A (553bp) | 553 |
| Cas3-MR1 | tgacggcggtgatgattaccatGAGGGATTGATTGGCATGATGG |  |  |
| Cas3-MF2 | ccatcatgccaatcaatccctcATGGTAATCATCACCGCCGTCA | *cas3* downstream flanking sequences B (571bp) | 571 |
| Cas3-MR2 | acagctagcgacgatatgtcGCAAGCCGACTTTCATTAAGCGT |  |  |
| Cas3-MF1 | ggaatctagaccttgagtcgAGCCCGGTACGATAGCCTTTG | Upstream and downstream homologous arm fusion fragment AB | 1124 |
| Cas3-MR2 | acagctagcgacgatatgtcGCAAGCCGACTTTCATTAAGCGT |  |  |
| plP12-TF | GACATATCGTCGCTAGCTGT | Linearized pLP12 vector | 3653 |
| plP12-TR | CGACTCAAGGTCTAGATTCC |  |  |
| pLP-UF | GACACAGTTGTAACTGGTCCA | pLP12-Cas3 recombinant plasmid | 1374 |
| pLP-UR | CAGGAACACTTAACGGCTGAC |  |  |
| Cas3-TF | GCATCACGTTGAGCCACAGCTT | Test the correct construction of *Cas3* gene knock-out strain Cas3-KO | 1347  /3915 |
| Cas3-TR | GCCCGTGAATGATGGAGTGTAAC |  |  |
| PBAD30-ZF | CTAGAGTCGACCTGCAGGCA | Linearized pBAD33-CM vector | 5529 |
| PBAD30-ZR | AGCTCGAATTCGCTAGCCCA |  |  |
| pBAD33CM-RP4-F2 | CGAATTGGGTACCAGCGCTT | Test the correct pBAD33-CM plasmid | 273 |
| pBAD33CM-RP4-R2 | TACCGTCGACGCCGGCCAGC |  |  |
| Cas3-RF | tgggctagcgaattcgagctAGGAGGAATTCACCGTTTACGTGTCGATA | *cas3* gene in *Salmonella* SE211-WT strain | 2718 |
| Cas3-RR | tgcctgcaggtcgactctagTTATTCCCCCATCATGCC |  |  |
| PBAD30-mcf-TF | CCATAAGATTAGCGGATCCTACCT | Test the correct construction of Complementary strain Comp/Cas3-KO | 2822 |
| PBAD30-mcf-TR | CTTCTCTCATCCGCCAAAACAG |  |  |
| 16S rRNA-F | CGGGGAGGAAGGTGTTGTG | Reference gene 16S rRNA for qPCR | 178 |
| 16S rRNA-R | GAGCCCGGGGATTTCACATC |  |  |
| Cas3-qF | CAGCTCAAGCGCCTTTTGTT | *cas3* gene expression detection for qPCR | 199 |
| Cas3-qR | TGGTTCTGCCAGCCGTTATT |  |  |
| Cse1-qF | GCATCCTTCGCATTGGTGAAC | *cse1* gene expression detection for qPCR | 112 |
| Cse1-qR | TGACGGAAGGTCTGATCCCT |  |  |
| Cse2-qF | TGGTAGTATTCCAGCGCCCAG | *cse2* gene expression detection for qPCR | 196 |
| Cse2-qR | CGGTAAAAACGCCGGATGAA |  |  |
| Cas7-qF | CCGTCTCCTGTGCATAGACC | *cas7* gene expression detection for qPCR | 139 |
| Cas7-qR | GAAAAAGGCACCGACCAACC |  |  |
| Cas5-qF | CAACAGCGCAGAACGCAAA | *cas5* gene expression detection for qPCR | 100 |
| Cas5-qR | TCTGGTTTTTCAGCTTCATGGG |  |  |
| Cas6-qF | CATCTGCCGGGATTTCTCTC | *cas6* gene expression detection for qPCR | 162 |
| Cas6-qR | CGTAAAACGCAGGGCGATAG |  |  |
| Cas1-qF | CCCTTCTCCCAGTCTTTCGG | *cas1* gene expression detection for qPCR | 190 |
| Cas1-qR | ACCTGCGGCTTAAAGTAGTCC |  |  |
| Cas2-qF | CGTCTGTTTTCACCCCAGGT | *cas2* gene expression detection for qPCR | 183 |
| Cas2-qR | ACGTCTTGCCGTCTGGTTAC |  |  |
| lsrA-qF | ACGCATGACCTTTCTACCGA | *lsrA* gene expression detection for qPCR | 140 |
| lsrA-qR | GCTAATACCGTCGCACCAC |  |  |
| lsrB-qF | GCAATGCAAAGAGGCGTGAA | *lsrB* gene expression detection for qPCR | 124 |
| lsrB-qR | GAGCGACCATCTCTACCAGC |  |  |
| lsrC-qF | AATCGGCTGGTTTACCCTGG | *lsrC* gene expression detection for qPCR | 207 |
| lsrC-qR | CCAATCTGCGAGGCAAACAC |  |  |
| lsrD-qF | ATATTTATGGCGGCTCCGGG | *lsrD* gene expression detection for qPCR | 172 |
| lsrD-qR | TGACGATGCAAACTGACGGA |  |  |
| lsrE-qF | CTACTGGCAGTGAACCCTGG | *lsrE* gene expression detection for qPCR | 267 |
| lsrE-qR | AGTAGTATCCCCGGCAACCT |  |  |
| lsrF-qF | GGCGAGGATTTTTAACCCCAA | *lsrF* gene expression detection for qPCR | 161 |
| lsrF-qr | TGCGTAATATGCCGCGAGTA |  |  |
| lsrG-qF | GCACGTTACGCTGGTTGAAA | *lsrG* gene expression detection for qPCR | 209 |
| lsrG-qR | AGTGTGGCGTTGTCTTGTGA |  |  |
| lsrK-qF | GGGATGTCGTTAAGCCACCA | *lsrK* gene expression detection for qPCR | 135 |
| lsrK-qR | AAAAGCTAGTGCGCTGGGAT |  |  |
| lsrR-qF | TTACGCTACAAGCGGCATCA | *lsrR* gene expression detection for qPCR | 215 |
| lsrR-qR | TGGTCTGCGCTTAGGAATCG |  |  |
| luxS-qF | GCGCATAAAGCCAGCAAACA | *luxS* gene expression detection for qPCR | 128 |
| luxS-qR | AAACGATGAACACCCCGCAT |  |  |
| sopE-qF | AGTCGGCATAGCACACTCATT | *sopE* gene expression detection for qPCR | 240 |
| sopE-qR | CTTAAGGAAATTGGCGAGGCT |  |  |
| spaM-qF | TTCCAGCTCGCTCCGTTTTT | *spaM* gene expression detection for qPCR | 120 |
| spaM-qR | AGACAGCTCAGTCGTGAGGA |  |  |
| spaN-qF | CGTGGCGTTATCGGCTACT | *spaN* gene expression detection for qPCR | 147 |
| spaN-qR | GGTTTGATTTCTGCGGAGGC |  |  |
| spaO-qF | GCAATAAACTTCCGCACGGG | *spaO* gene expression detection for qPCR | 179 |
| spaO-qR | GCGGCCTGTGGTTTGAATATC |  |  |
| spaP-qF | TAAGGTCCAGCCATCAAGCG | *spaP* gene expression detection for qPCR | 120 |
| spaP-qR | GTCGTCGACCTGGTGGTATC |  |  |
| spaQ-qF | CCTTAGCCAACGCCAGGAAT | *spaQ* gene expression detection for qPCR | 133 |
| spaQ-qR | TTACAGGAACAGACGCTGCC |  |  |
| spaR-qF | ACCAGACCGCCGTTTTGTAA | *spaR* gene expression detection for qPCR | 150 |
| spaR-qR | TATGCATGCGCTGGGTTGTA |  |  |
| spaS-qF | GTGGGGTTGGCAACAATCAG | *spaS* gene expression detection for qPCR | 129 |
| spaS-qR | GCGTGAAATGAAGGAGCAGG |  |  |

Note: The sequences with yellow and purples shadow respectively represent the upstream and downstream homologous arms of pLP12 vector. The sequences with green and light blue shadow respectively respresent upstream and downstream flanking homologous arms of *cas3* gene. The sequences with blue and red shadow respectively represent the upstream and downstream homologous arms of pBAD33-CM vector. The sequence with grey shadow represents Ribosome Bind Site (RBS).
