# Supplementary material for: CRISPR-cas3 of Salmonella Upregulates Bacterial Biofilm Formation and Virulence to Host Cells by Targeting Quorum-Sensing Systems
Source: Pathogens. 2020 Jan 10;9(1):53. doi: 10.3390/pathogens9010053 (PMC7168661; doi:10.3390/pathogens9010053)
Supplement: Supplementary file 1 [file pathogens-09-00053-s001.zip › fig. S1 biofilm and cell infection.docx]

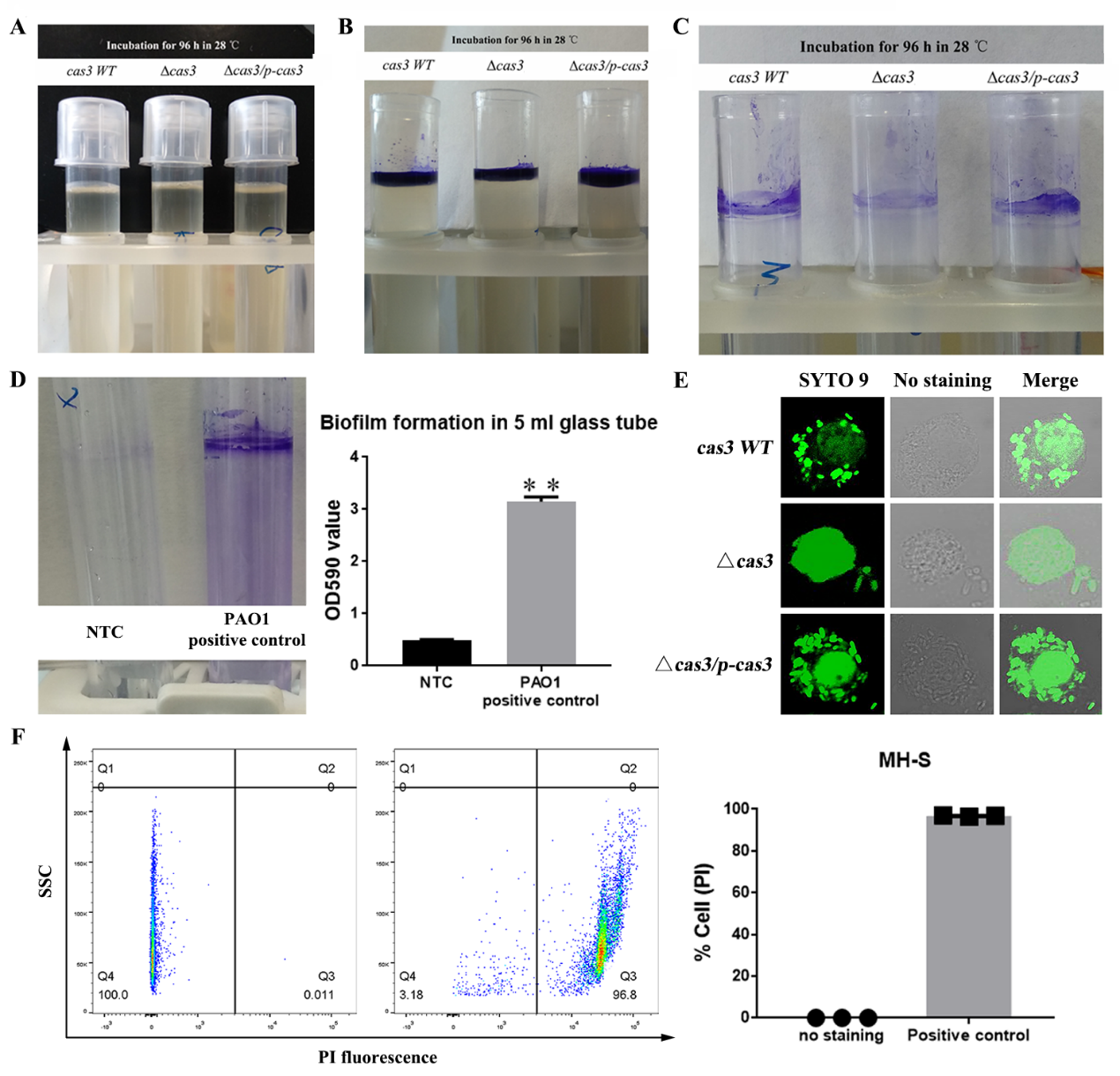


**Fig. S1** Impact of *cas3* on biofilm formation and cell infection of *Salmonella*. (A-D) Biofilm formation of *cas3* WT, Δ*cas3*, Δ*cas3/p-cas3* and PAO1 in LB broth was visualized after 96 h of incubation at 28 ^o^C as a floating pellicle at the air-broth interface, The biofilm rings of bacteria adhered to the tube wall were stained by crystal violet and washed by distilled water, then quantified by crystal violet, related to Fig. 2C-D. (E) Fluorescence images of the MH-S cells and intracellular live *Salmonella* stained with SYTO^®^ 9 green-fluorescent nucleic acid stains after 4 h post-infection of *cas3* WT, Δ*cas3* and Δ*cas3/p-cas3* strains respectively. (F) an unstained control and a positive control of MH-S cell survival assay was detected, related to Fig. 3F.
