# Supplementary material for: CRISPR-cas3 of Salmonella Upregulates Bacterial Biofilm Formation and Virulence to Host Cells by Targeting Quorum-Sensing Systems
Source: Pathogens. 2020 Jan 10;9(1):53. doi: 10.3390/pathogens9010053 (PMC7168661; doi:10.3390/pathogens9010053)
Supplement: Supplementary file 1 [file pathogens-09-00053-s001.zip › Fig. S2 DEGs.docx]

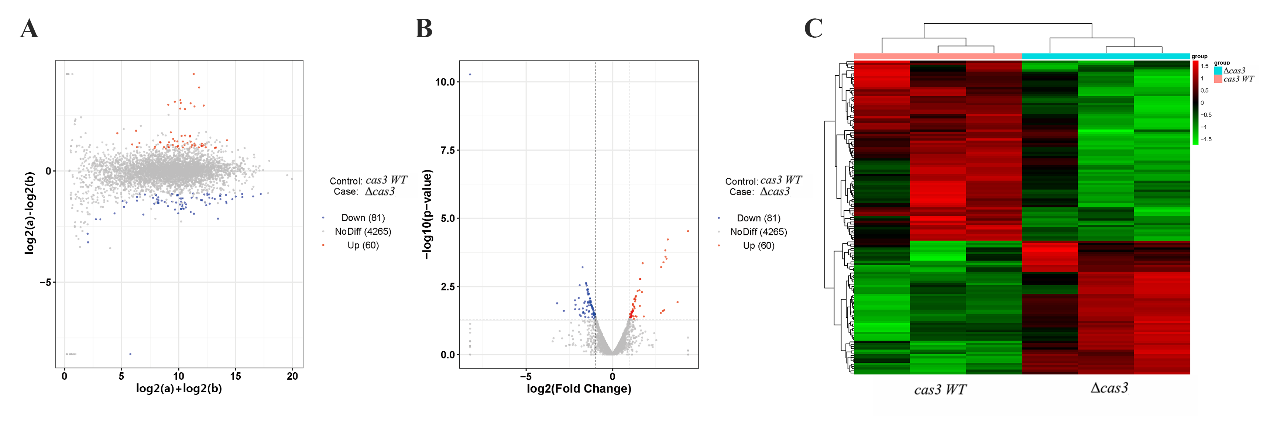


**Fig. S2** Differentially expressed genes (DEGs) between Δ*cas3* and *cas3* WT strains. (A-B) Comparison of expression levels between the Δ*cas3* and *cas3* WT genes. All genes were classified into three classes. The red dots indicate the Δ*cas3* strains up-regulated genes (60 genes), the blue dots indicated the down-regulated genes (81 genes) and the grey dots represent the non-DEGs. (A) MA Plot. a: the gene expression of Δ*cas3*, b: the gene expression of *cas3* WT. (B) Volcano Plot. The vertical dashed lines indicate the 2 folds expression change (Δ*cas3* vs *cas3* WT strain). The transverse dashed lines indicate the p-value=0.05. (C) Union hierarchical cluster analysis of transcript expression profiles with DEGs. X axis represents each sample. Y axis represents DEGs. Coloring indicate fold change (high: red, low: blue).
