# Supplementary material for: CRISPR-cas3 of Salmonella Upregulates Bacterial Biofilm Formation and Virulence to Host Cells by Targeting Quorum-Sensing Systems
Source: Pathogens. 2020 Jan 10;9(1):53. doi: 10.3390/pathogens9010053 (PMC7168661; doi:10.3390/pathogens9010053)
Supplement: Supplementary file 1 [file pathogens-09-00053-s001.zip › Fig. S3 KEGG.docx]

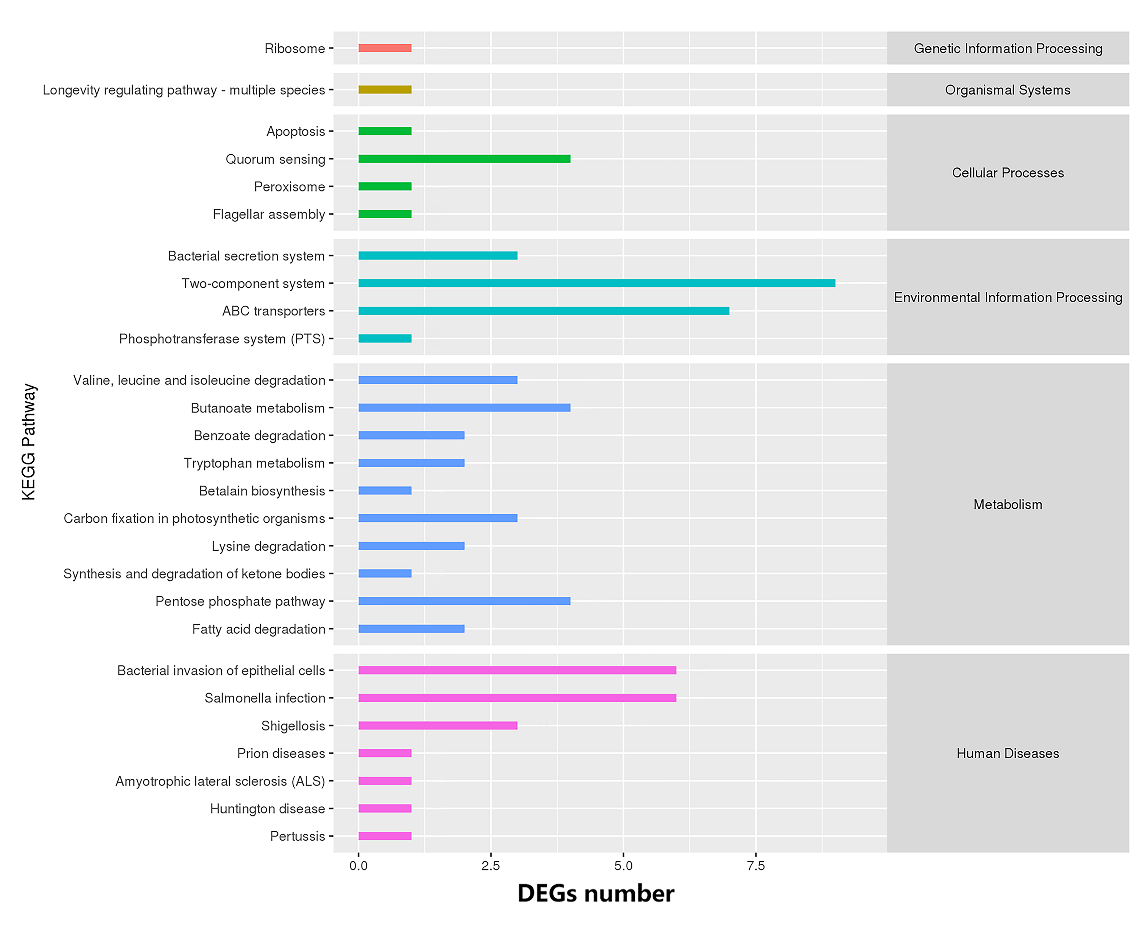

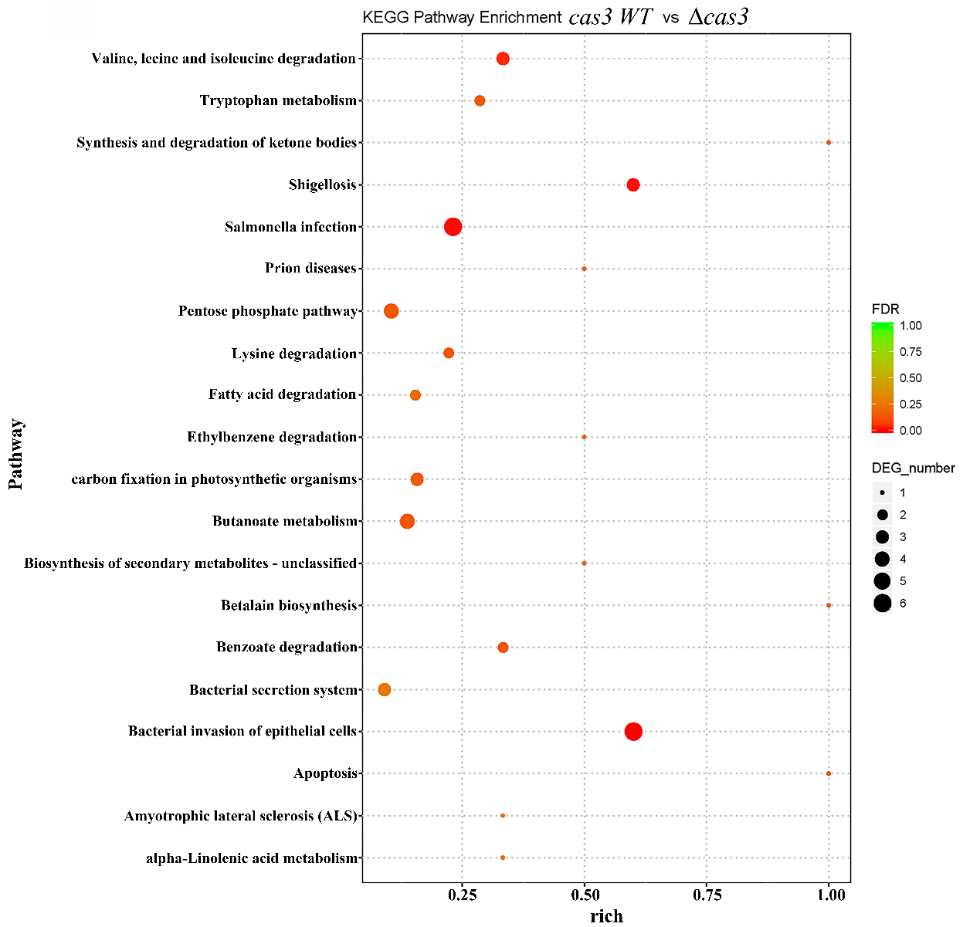


**B**

**A**

**Fig. S3** Kyoto Encyclopedia of Genes and Genomes (KEGG) pathway classification and enrichment of DEGs. (B) X axis represents enrichment factor. Y axis represents pathway name. Coloring indicate FDR value (high: green, low: red), a p-value corrected by multiple hypothesis tests. The lower FDR value indicates the more significant enriched. Point size indicates DEGs number (more: big, less: small).
