# Supplementary material for: CRISPR-cas3 of Salmonella Upregulates Bacterial Biofilm Formation and Virulence to Host Cells by Targeting Quorum-Sensing Systems
Source: Pathogens. 2020 Jan 10;9(1):53. doi: 10.3390/pathogens9010053 (PMC7168661; doi:10.3390/pathogens9010053)
Supplement: Supplementary file 1 [file pathogens-09-00053-s001.zip › Table S1-LD50.docx]

Table S1. Mortality data for chickens following oral infection by *Salmonella*

| **Group** | **Dose (CFU)** | **Animal number** | **Death number** | **Death rate P (%)** | **LD_50_ (CFU/mL)** |
| --- | --- | --- | --- | --- | --- |
| 0.9% NaCl | 0 | 10 | 0 | 0 | - |
| *cas3* WT | 4.7$\times{10}^{11}$ | 10 | 7 | 70 | (9.378$\pm2.075)\times{10}^{10}$ |
|  | 4.7$\times{10}^{10}$ | 10 | 3 | 30 |  |
|  | 4.7$\times{10}^{9}$ | 10 | 2 | 20 |  |
|  | 4.7$\times{10}^{8}$ | 10 | 0 | 0 |  |
| Δ*cas3* | 3.22$\times{10}^{11}$ | 10 | 3 | 30 | (4.029$\pm0.028)\times{10}^{11}$ |
|  | 3.22$\times{10}^{10}$ | 10 | 1 | 10 |  |
|  | 3.22$\times{10}^{9}$ | 10 | 0 | 0 |  |
|  | 3.22$\times{10}^{8}$ | 10 | 0 | 0 |  |
| Δ*cas3/p-cas3* | 3.07$\times{10}^{11}$ | 10 | 7 | 70 | (3.07$\pm2.944)\times{10}^{10}$ |
|  | 3.07$\times{10}^{10}$ | 10 | 4 | 40 |  |
|  | 3.07$\times{10}^{9}$ | 10 | 3 | 30 |  |
|  | 3.07$\times{10}^{8}$ | 10 | 1 | 10 |  |
