# Supplementary material for: CRISPR-cas3 of Salmonella Upregulates Bacterial Biofilm Formation and Virulence to Host Cells by Targeting Quorum-Sensing Systems
Source: Pathogens. 2020 Jan 10;9(1):53. doi: 10.3390/pathogens9010053 (PMC7168661; doi:10.3390/pathogens9010053)
Supplement: Supplementary file 1 [file pathogens-09-00053-s001.zip › Table S2-T3SS.docx]

Table S2. The differentially expressed genes (DEGs) located at SPI-1 and related to SPI-1-T3SS in *Salmonella* Δ*cas3* strain versus *cas3* WT strain

| **Gene** | **Protein** | **Supply** | **Fold change (****Δ*cas3*/*****cas3* WT)** | **p-value** | **Function** |
| --- | --- | --- | --- | --- | --- |
| *invF* | AraC family transcriptional regulator InvF | Invasion protein InvF | -2.72 | 0.004 | Transcriptional regulator required for the expression of several genes encoding type III secretion system SPI1 effector proteins. The interaction with SicA is necessary for the activation of *sigDE* (*sopB*, *pipC*), *sicA*, *sipBCDA*, and *sopE* ^[1-4](#_ENREF_1)^. |
| *invA* | type III secretion system export app aratus protein InvA | virulence associated secretory protein, Invasion protein InvA | -2.11 | 0.032 | Involved in the invasion of the cells of the intestinal epithelium. Could be involved in the translocation of the InvE protein. |
| *invE* | type III secretion system gatekeeper InvE | invasion protein | -2.07 | 0.034 | Involved in the triggering of intracellular events that lead to microbial internalization. These events include increase in calcium level, redistribution of actin microfilaments, and changes in the normal structure of the microvilli. Encoded within the type III secretion system (SPI-1 TTSS), it is essential for the translocation of protein effectors into host cells. Forms a complex with SipB and SipC in the presence of their chaperone SicA. Positively regulates the secretion of SPI-1 TTSS effector proteins SipB, SipC and SipD and negatively influences the secretion of SipA, SopA and SptP ^[5](#_ENREF_5), [6](#_ENREF_6)^. |
| *invH* | invasion lipoprotein InvH |  | -1.73 | 0.120 |  |
| *invG* | outer membrane secretin precursor |  | -1.98 | 0.048 | - |
| *orgA* | oxygen-regulated invasion protein OrgA | needle complex assembly protein, | -2.41 | 0.012 | Oxygen-regulated protein required for bacterial internalization. |
| *orgB* | oxygen-regulated invasion protein OrgB | needle complex export protein | -2.15 | 0.030 | Oxygen-regulated protein required for bacterial internalization ^[7](#_ENREF_7)^. |
| *orgC* | type III secretion system effector protein OrgC |  | -2.34 | 0.016 |  |
| *prgJ* | type III secretion system inner rod protein PrgJ | needle complex minor subunit | -2.90 | 0.002 | Required for invasion of epithelial cells ^[8](#_ENREF_8)^. |
| *prgI* | type III secretion system needle complex protein PrgI | needle complex major subunit | -2.67 | 0.004 | Required for invasion of epithelial cells ^[7](#_ENREF_7)^. |
| *prgK* | type III secretion system inner membrane ring lipoprotein PrgK | needle complex inner membrane lipoprotein | -2.41 | 0.011 | Required for invasion of epithelial cells. Could be involved in protein secretion ^[7](#_ENREF_7), [9](#_ENREF_9)^. |
| *prgH* | cell invasion protein |  | -2.21 | 0.022 |  |
| *sptP* | SPI-1 type III secretion system effector GTPase-activating protein SptP | tyrosine phosphatase, Secreted effector protein SptP | -2.59 | 0.006 | Effector proteins function to alter host cell physiology and promote bacterial survival in host tissues. This protein includes tyrosine phosphatase and GTPase activating protein (GAP) activities. After bacterial internalization, GAP mediates the reversal of the cytoskeletal changes induced by SopE. This function is independent of its tyrosine phosphatase activity, which remains unclear ^[10-12](#_ENREF_10)^. |
| *sipA* | SPI-1 type III secretion system effector SipA | pathogenicity island 1 effector protein, Cell invasion protein SipA | -2.21 | 0.020 | Actin-binding protein that interferes with host cell actin cytoskeleton. It stimulates actin polymerization and counteracts F-actin destabilizing proteins. Potentiates SipC activity; both are required for an efficient bacterial internalization (By similarity). |
| *sipD* | SPI-1 type III secretion system needle tip complex protein SipD | A Chain A, Sipd From *Salmonella* Typhimurium, Cell invasion protein SipD | -2.05 | 0.034 | Required for translocation of effector proteins via the type III secretion system SPI-1, which is essential for an efficient bacterial internalization. Probably acts by modulating the secretion of SipA, SipB, and SipC ^[13](#_ENREF_13), [14](#_ENREF_14)^. |
| *sipC* | SPI-1 type III secretion system needle tip complex protein SipC | pathogenicity island 1 effector protein, Cell invasion protein SipC | -2.04 | 0.032 | Actin-binding protein that interferes with host cell actin cytoskeleton. Nucleates actin polymerization and condensates actin filaments into cables (bundling). SipA potenciates SipC activity and both are required for an efficient bacterial internalization by the host cell ^[15](#_ENREF_15)^. |
| *sipB* | cell invasion protein |  | -1.99 | 0.038 | - |
| *spaM* | SPI-1 type III secretion system protein SpaM | needle complex assembly protein, Surface presentation of antigens protein SpaM | -2.68 | 0.006 | Involved in a secretory pathway responsible for the surface presentation of determinants needed for the entry of *Salmonella* species into mammalian cells. |
| *spaS* | SPI-1 type III secretion system export apparatus protein SpaS | type III secretion system protein SpaS, Surface presentation of antigens protein SpaS | -2.59 | 0.016 | Involved in a secretory pathway responsible for the surface presentation of determinants needed for the entry of *Salmonella* species into mammalian cells. |
| *spaQ* | SPI-1 type III secretion system export apparatus protein SpaQ | virulence associated secretory protein, Surface presentation of antigens protein SpaQ | -2.56 | 0.025 | Involved in a secretory pathway responsible for the surface presentation of determinants needed for the entry of *Salmonella* species into mammalian cells. |
| *spaO* | SPI-1 type III secretion system protein SpaO | surface presentation of antigens protein SpaO | -2.49 | 0.009 | Involved in a secretory pathway responsible for the surface presentation of determinants needed for the entry of *Salmonella* species into mammalian cells. |
| *spaK* | SPI-1 type III secretion system chaperone SpaK | type III secretion system chaperone SpaK, Surface presentation of antigens protein SpaK | -2.47 | 0.009 | Involved in a secretory pathway responsible for the surface presentation of determinants needed for the entry of *Salmonella* species into mammalian cells. Chaperone specialized in the storage of effectors within the bacterial cytoplasm, maintaining them in a secretion-competent state, and allowing their immediate delivery to target cells upon contact of the bacterium with the host cells. Has been shown to chaperone SopA, SopE, SopE2 and SipA ^[16](#_ENREF_16), [17](#_ENREF_17)^. |
| *spaN* | surface presentation of antigens protein |  | -2.39 | 0.012 |  |
| *spaP* | SPI-1 type III secretion system export apparatus protein SpaP | surface presentation of antigens protein SpaP | -2.35 | 0.016 | Involved in a secretory pathway responsible for the surface presentation of determinants needed for the entry of *Salmonella* species into mammalian cells. |
| *spaL* | FliI/YscN family ATPase | ATP synthase SpaL | -2.29 | 0.017 | Necessary for efficient entry of S.typhimurium into cultured epithelial cells. Probable catalytic subunit of a protein translocase. May energize the protein export apparatus encoded in the inv locus which is required for the surface presentation of determinants needed for the entry of *Salmonella* species into mammalian cells. |
| *spaR* | secretory protein (associated with virulence) |  | -2.68 | 0.086 | - |
| *pagO* | PhoPQ-activated integral membrane protein |  | -2.05 | 0.040 | - |
| *pagN* | Outer membrane protein, PagN | adhesin/invasin protein PagN | -2.01 | 0.035 | Haemagglutinin that facilitates the adhesion to and invasion of epithelial mammalian cells. Utilizes heparinated proteoglycan as a receptor to successfully invade host cells ^[18](#_ENREF_18), [19](#_ENREF_19)^. |
| *pagD* | virulence protein PAGD precursor |  | -1.71 | 0.123 | + |
| *pagC* | virulence membrane protein PagC |  | -1.24 | 0.497 | - |
| *pagP* | phospholipid:lipid A palmitoyltransferase |  | -1.33 | 0.797 | + |
| *pagK* | phage encoded PagK |  | -1.04 | 0.846 | - |
| *sicP* | chaperone protein SicP | chaperone | -2.82 | 0.003 | Molecular chaperone required for sptP stabilization and secretion ^[20](#_ENREF_20)^. |
| *sicA* | type III secretion system translocator chaperone SicA | Chaperone protein SicA | -2.19 | 0.022 | Type III secretion-associated chaperone required for SipB and SipC stabilization. Prevents premature association of SipB with SipC, which may lead to their targeting for degradation. Along with InvF, required for transcription activation of sigDE (sopB pipC), sicAsipBCDA, and sopE. |
| *sopE* | type III secretion system guanine nucleotide exchange factor SopE | type III secretion system, secreted effector protein SopE | -2.25 | 0.017 | Activator for both CDC42 and RAC1 by directly engaging these Rho GTPases and acting as potent guanine nucleotide exchange factor (GEF). This activation results in actin cytoskeleton rearrangements and stimulates membrane ruffling, promoting bacterial entry into non-phagocytic cells. |
| *sopE2* | SPI-1 type III secretion system guanine nucleotide exchange factor SopE2 | invasion-associated secreted effector protein (sopE2) | -2.08 | 0.029 | Activator for CDC42 by directly engaging this Rho GTPase and acting as potent guanine nucleotide exchange factor (GEF). This activation results in actin cytoskeleton rearrangements and stimulates membrane ruffling, promoting bacterial entry into non-phagocytic cells. Also activates NF-kB, p38 and ERK kinases, which are known to be involved in the induction of IL-8 expression. Chaperone InvB is required for secretion, translocation and stabilization of intracellular levels of sopE2 ^[21](#_ENREF_21), [22](#_ENREF_22)^. |
| *steB* |  | dipicolinate reductase | -2.67 | 0.012 |  |

References

1. Eichelberg K, Galan JE. Differential regulation of Salmonella typhimurium type III secreted proteins by pathogenicity island 1 (SPI-1)-encoded transcriptional activators InvF and hilA. Infection and immunity 1999; 67:4099-105.

2. Darwin KH, Miller VL. InvF is required for expression of genes encoding proteins secreted by the SPI1 type III secretion apparatus in Salmonella typhimurium. J Bacteriol 1999; 181:4949-54.

3. Darwin KH, Miller VL. The putative invasion protein chaperone SicA acts together with InvF to activate the expression of Salmonella typhimurium virulence genes. Molecular microbiology 2000; 35:949-60.

4. Darwin KH, Miller VL. Type III secretion chaperone-dependent regulation: activation of virulence genes by SicA and InvF in Salmonella typhimurium. The EMBO journal 2001; 20:1850-62.

5. Ginocchio C, Pace J, Galan JE. Identification and molecular characterization of a Salmonella typhimurium gene involved in triggering the internalization of salmonellae into cultured epithelial cells. Proceedings of the National Academy of Sciences of the United States of America 1992; 89:5976-80.

6. Kubori T, Galan JE. Salmonella type III secretion-associated protein InvE controls translocation of effector proteins into host cells. J Bacteriol 2002; 184:4699-708.

7. Klein JR, Fahlen TF, Jones BD. Transcriptional organization and function of invasion genes within Salmonella enterica serovar Typhimurium pathogenicity island 1, including the prgH, prgI, prgJ, prgK, orgA, orgB, and orgC genes. Infection and immunity 2000; 68:3368-76.

8. Lefebre MD, Galan JE. The inner rod protein controls substrate switching and needle length in a Salmonella type III secretion system. Proceedings of the National Academy of Sciences of the United States of America 2014; 111:817-22.

9. Bergeron JRC, Brockerman JA, Vuckovic M, Deng W, Okon M, Finlay BB, et al. Characterization of the two conformations adopted by the T3SS inner-membrane protein PrgK. Protein science : a publication of the Protein Society 2018; 27:1680-91.

10. Kaniga K, Uralil J, Bliska JB, Galan JE. A secreted protein tyrosine phosphatase with modular effector domains in the bacterial pathogen Salmonella typhimurium. Molecular microbiology 1996; 21:633-41.

11. Fu Y, Galan JE. A salmonella protein antagonizes Rac-1 and Cdc42 to mediate host-cell recovery after bacterial invasion. Nature 1999; 401:293-7.

12. Stebbins CE, Galan JE. Modulation of host signaling by a bacterial mimic: structure of the Salmonella effector SptP bound to Rac1. Molecular cell 2000; 6:1449-60.

13. Kaniga K, Trollinger D, Galan JE. Identification of two targets of the type III protein secretion system encoded by the inv and spa loci of Salmonella typhimurium that have homology to the Shigella IpaD and IpaA proteins. J Bacteriol 1995; 177:7078-85.

14. Collazo CM, Galan JE. The invasion-associated type III system of Salmonella typhimurium directs the translocation of Sip proteins into the host cell. Molecular microbiology 1997; 24:747-56.

15. Hayward RD, Koronakis V. Direct nucleation and bundling of actin by the SipC protein of invasive Salmonella. The EMBO journal 1999; 18:4926-34.

16. Higashide W, Zhou D. The first 45 amino acids of SopA are necessary for InvB binding and SPI-1 secretion. J Bacteriol 2006; 188:2411-20.

17. Lilic M, Vujanac M, Stebbins CE. A common structural motif in the binding of virulence factors to bacterial secretion chaperones. Molecular cell 2006; 21:653-64.

18. Lambert MA, Smith SG. The PagN protein of Salmonella enterica serovar Typhimurium is an adhesin and invasin. BMC microbiology 2008; 8:142.

19. Lambert MA, Smith SG. The PagN protein mediates invasion via interaction with proteoglycan. FEMS microbiology letters 2009; 297:209-16.

20. Fu Y, Galan JE. Identification of a specific chaperone for SptP, a substrate of the centisome 63 type III secretion system of Salmonella typhimurium. J Bacteriol 1998; 180:3393-9.

21. Stender S, Friebel A, Linder S, Rohde M, Mirold S, Hardt WD. Identification of SopE2 from Salmonella typhimurium, a conserved guanine nucleotide exchange factor for Cdc42 of the host cell. Molecular microbiology 2000; 36:1206-21.

22. Huang FC, Werne A, Li Q, Galyov EE, Walker WA, Cherayil BJ. Cooperative interactions between flagellin and SopE2 in the epithelial interleukin-8 response to Salmonella enterica serovar typhimurium infection. Infection and immunity 2004; 72:5052-62.
